# Real-world routine diagnostic molecular analysis for *TP53* mutational status is recommended over p53 immunohistochemistry in B-cell lymphomas

**DOI:** 10.1007/s00428-023-03676-6

**Published:** 2023-10-18

**Authors:** Lorraine M. de Haan, Ruben A. L. de Groen, Fleur A. de Groot, Troy Noordenbos, Tom van Wezel, Ronald van Eijk, Dina Ruano, Arjan Diepstra, Lianne Koens, Alina Nicolae-Cristea, Wietske C. E. den Hartog, Valeska Terpstra, Els Ahsmann, Tim J. A. Dekker, Aniko Sijs-Szabo, Hendrik Veelken, Arjen H. G. Cleven, Patty M. Jansen, Joost S. P. Vermaat

**Affiliations:** 1 Department of Pathology, Leiden University Medical Center, L1-Q, P.O. box 9600, 2300RC Leiden, The Netherlands; 2 Department of Hematology, Leiden University Medical Center, Leiden, The Netherlands; 3 Department of Pathology, University Medical Center Groningen, Groningen, The Netherlands; 4 Department of Pathology, Amsterdam University Medical Center, Amsterdam, The Netherlands; 5 Department of Pathology, Haga Hospital, The Hague, The Netherlands; 6 Department of Pathology, Alrijne Hospital, Leiden, The Netherlands; 7 Department of Pathology, Haaglanden Medical Centrum, The Hague, The Netherlands; 8 Department of Pathology, Groene Hart Ziekenhuis, Gouda, The Netherlands

**Keywords:** B-cell lymphoma, Molecular diagnostics, Targeted therapy, Immunohistochemistry, Hematopathology

## Abstract

Previous studies in patients with mature B-cell lymphomas (MBCL) have shown that pathogenic *TP53* aberrations are associated with inferior chemotherapeutic efficacy and survival outcomes. In solid malignancies, p53 immunohistochemistry is commonly used as a surrogate marker to assess *TP53* mutations, but this correlation is not yet well-established in lymphomas. This study evaluated the accuracy of p53 immunohistochemistry as a surrogate marker for *TP53* mutational analysis in a large real-world patient cohort of 354 MBCL patients within routine diagnostic practice. For each case, p53 IHC was assigned to one of three categories: wild type (staining 1–50% of tumor cells with variable nuclear staining), abnormal complete absence or abnormal overexpression (strong and diffuse staining > 50% of tumor cells). Pathogenic variants of *TP53* were identified with a targeted next generation sequencing (tNGS) panel. Wild type p53 expression was observed in 267 cases (75.4%), complete absence in twenty cases (5.7%) and the overexpression pattern in 67 cases (18.9%). tNGS identified a pathogenic *TP53* mutation in 102 patients (29%). The overall accuracy of p53 IHC was 84.5% (95% CI 80.3–88.1), with a robust specificity of 92.1% (95% CI 88.0- 95.1), but a low sensitivity of 65.7% (95% CI 55.7–74.8). These results suggest that the performance of p53 IHC is insufficient as a surrogate marker for *TP53* mutations in our real-world routine diagnostic workup of MBCL patients. By using p53 immunohistochemistry alone, there is a significant risk a *TP53* mutation will be missed, resulting in misevaluation of a high-risk patient. Therefore, molecular analysis is recommended in all MBCL patients, especially for further development of risk-directed therapies based on *TP53* mutation status.

**Supplementary Information:**

The online version contains supplementary material available at 10.1007/s00428-023-03676-6.

## Introduction

Lymphomas encompass more than 80 different types of malignancies, each distinguished by unique clinical, morphological, immunohistochemical, molecular, and cytogenetic characteristics [1]. The p53 protein encoded by the *TP53* gene is an important tumor suppressor that mediates cell-cycle arrest, DNA repair, transcription, signalling, metabolism, apoptosis, and autophagy [2, 3]. In human cancers, nonsynonymous or missense mutations in *TP53*, often accompanied by loss of heterozygosity, is the most common mechanism leading to altered p53 sequence and structure. This is consistent with the so called ‘two-hit’ hypothesis; inactivation of both copies of a tumor suppressor gene are required for dysfunction, often resulting in gain-of-function (pro-oncogenic) or loss-of-function (decreased tumor suppression) [4]. The overall prevalence of *TP53* mutations in cancer is around 50%, reaching 100% in some carcinomas such as high-grade ovarian cancer [4–6]. In contrast, homozygous deletions of *TP53* are exceedingly rare (0.2%) [7]. *TP53* mutations resulting in p53 dysfunction are less frequent in lymphoid malignancies than in other types of cancer [2, 8]. In mature B-cell lymphomas (MBCL) the frequency varies across different subtypes, ranging from approximately 10% in chronic lymphocytic leukemia (CLL) to 25% in diffuse large B-cell lymphoma not otherwise specified (DLBCL, NOS) and Burkitt lymphoma (BL). Several studies in MBCL patients have shown that pathogenic *TP53* aberrations are associated with inferior therapeutic efficacy and survival outcomes [3, 9–11]. *TP53* mutational status has a central role in the current treatment algorithms for both CLL and mantle cell lymphoma (MCL). In *TP53*-aberrant CLL/MCL patients, therapy aims to block the B-cell receptor pathway with Bruton tyrosine kinase (BTK) inhibitors or B-cell lymphoma-2 (bcl2) inhibitors, which act independently of the *TP53* pathway as opposed to treatment with cytotoxic agents [9, 10].

DLBCL, NOS is the most prevalent subtype of MBCL and characterized by a poor prognosis, aggressive disease course, and significant genetic heterogeneity. The standard first-line therapy for DLBCL, NOS is the ‘one-size-fits-all’ immunochemotherapy regimen R-CHOP (rituximab, cyclophosphamide, doxorubicin, vincristine, prednisone). While this treatment can cure a proportion of patients, approximately 40% of patients develop disease recurrence and require additional therapy [12]. While *TP53* is at present only used as a therapy stratifier in MCL and CLL, there are multiple studies that have shown that pathogenic *TP53* aberrations are associated with inferior therapeutic efficacy and survival outcomes in other mature B-cell lymphomas as well, most notably in (D)LBCL[3, 11]. In DLBCL, NOS but also in other lymphomas, there is an urgent need for further development of targeted and risk-directed therapies that improve clinical outcomes by selecting the most optimal treatment for each patient based on intrinsic tumor factors. *TP53* mutational status has the potential to provide important prognostic and predictive information if reliable assessment can be achieved in routine clinical practice.

Regardless of the effect of *TP53* mutational status on the clinical course, targeted next generation sequencing (tNGS) is currently not standard practice in the diagnostic workup of lymphomas in most centers. In solid tumors, p53 immunohistochemistry (IHC) is an accurate and frequently used surrogate marker to assess *TP53* mutations. P53 IHC is widely available, interpretable by a pathologist, faster and relatively inexpensive compared to tNGS. Both overexpression or complete absence of p53 expression may indicate a pathogenic *TP53* mutation or deletion [13–17]. Gain-of-function *TP53* aberrations accompanied by loss of the tumor-suppressive function typically arise from missense mutations or in-frame deletions, disrupting MDM2 mediated ubiquitin degradation of p53, causing it to accumulate in the tumor cells leading to overexpression. Loss-of-function is usually caused by nonsense mutations, splicing mutations, or frameshifts, resulting in a premature stop gain and therefore no translated protein and IHC expression [18, 19]. Given the clinical impact of pathogenic *TP53* mutations, p53 IHC may be a valuable marker in MBCL diagnostics.

Since in solid tumors p53 IHC is an accurate and frequently used surrogate marker to assess *TP53* mutations, p53 IHC is also frequently used for lymphomas, while in this entity the performance of p53 IHC has currently not been definitively established. Studies in this area are lacking, particularly in indolent B-cell lymphomas (IBCL) and have primarily centred on (D)LBCL and MCL, rather than the broader MBCL population, thus not reflecting real-life everyday diagnostics [2, 3, 20]. With the implementation of tNGS in our center since 2017 for routine daily diagnostic procedures for MBCLs, we were in the unique setting to directly compare p53 IHC with *TP53* mutational analyses for many years. Therefore, in this retrospective study the diagnostic accuracy of p53 IHC as a surrogate marker for *TP53* mutational tNGS analysis was evaluated in a large real-world cohort of various MBCL subtypes.

## Methods

### Patient selection

For this retrospective study 354 patients diagnosed with BCL between 2017–2022 were selected. The cases were diagnosed according to the revised fourth edition WHO classification (2016) and included (diffuse) large B-cell lymphoma ((D)LBCL), mantle cell lymphoma (MCL) and indolent B-cell lymphoma (IBCL). The study was conducted in accordance with the Dutch Code for Proper Secondary Use of Human Tissue, the local institutional board requirements, and the revised Declaration of Helsinki (2008). Approval with a waiver of consent was obtained from the LUMC's medical ethics committee (B16.048).

### Immunohistochemistry and *in situ* hybridization

**Fig. 1:**
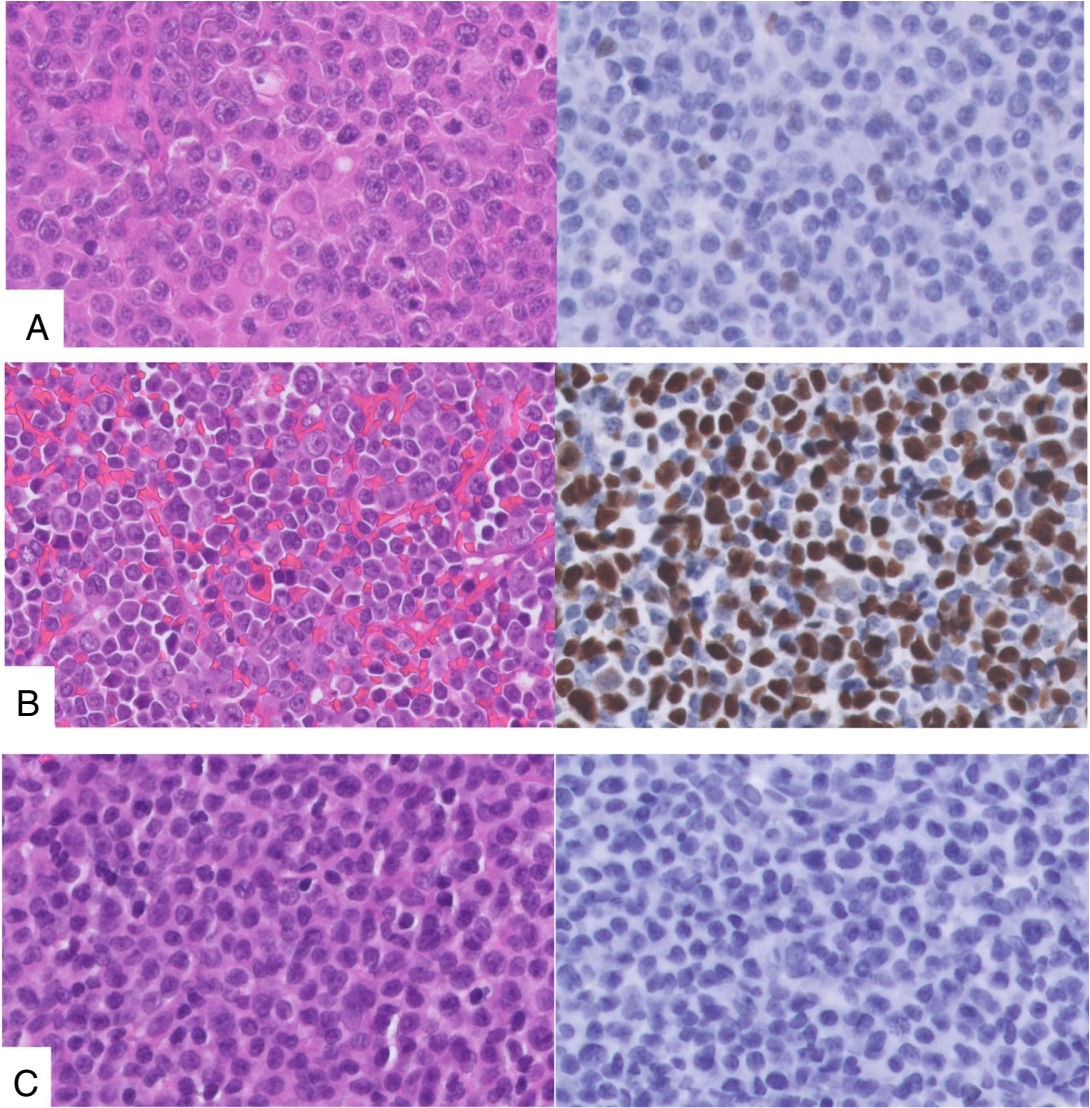
Representative microscopy of p53 immunohistochemistry staining patterns. **A** Diffuse large B-cell lymphoma, not otherwise specified (hematoxylin & Eosin × 80) with P53 wild type pattern, × 80. **B** Diffuse large B-cell lymphoma, not otherwise specified (hematoxylin & Eosin × 80) with P53 abnormal overexpression pattern, × 80. **C** Mantle cell lymphoma (hematoxylin & Eosin × 80) with P53 abnormal deletion pattern, × 80

A representative formalin-fixed paraffin-embedded (FFPE) tumor block was selected for each case, and three µm thin tissue sections were prepared**.** Tumor cell percentage was at least 20%, and for most cases more than 60%. The slides were stained for p53 antibody expression using the Dako Auto Stainer Link 48 (Dako Omnis, Monoclonal Mouse Anti Human, p53 clone DO-7, Glostrup, Denmark), according to standard procedures and as previously reported by our institute for endometrial carcinoma [13]. The p53 staining patterns were classified as wild type or abnormal, with cut-offs used in our daily diagnostics and as previously published in lymphoma patients[3, 21]**.** Wild type expression was defined as p53 expression in 1–50% of tumor nuclei with variable nuclear staining intensity (including weak, moderate, and strong). Abnormal p53 expression was defined as strong diffuse positive p53 expression in > 50% of the tumor nuclei (abnormal overexpression) or complete absence of p53 staining with a positive internal and/or external control (abnormal null mutant). Representative examples of the staining categories are shown in Fig. 1. All p53 IHC slides were independently scored by two hematopathologists (LMH and PMJ) who were blinded to the clinical and molecular data. All discordant cases were discussed at a consensus meeting attended by both pathologists where a definitive category was assigned. As described before, according to standard diagnostic lymphoma workup for LBCL, *MYC,* and when positive*, BCL2,* and *BCL6* rearrangements were analysed with fluorescence in situ hybridization (FISH), using break-apart probes [22]. The Epstein-Barr virus (EBV) status was determined using EBV-encoded RNA in situ hybridization (ISH).

### Targeted next-generation sequencing

DNA alterations in *TP53* were investigated using two tNGS panels. Only 10% (n = 36) of the samples were sequenced using amplicons depicted in online resource 1a, while the majority (n = 318, 90%) were sequenced using amplicons depicted in online resource 1b. Libraries prepared with the tNGS panels were sequenced using the Ion GeneStudio™ S5 System and the sequenced reads were mapped against the human reference genome (GRCh37/hg19) using iontorrent aligner (TMAP) and variant caller (TVC) using default parameters for somatic variant calling. Only variants with a predefined minimum coverage of 100 reads and variant allele frequency (VAF) of 0.10 (10%) were considered. Subsequently interpretation of the variants was done using Franklin genoox and GenomeNexus [23, 24], aggregating data from all public databases (including Cosmic, Clinvar and *TP53* databases) and classified into class 1 (benign), class 2 (likely benign), class 3 (unknown significance), class 4 (likely pathogenic), or class 5 (pathogenic) [25]. Variants classified as class 4 and 5 were selected for further analysis.

### Statistical analysis

The statistical analysis was conducted with RStudio (version 4.2.1). The inter-rater reliability was assessed using Cohen's kappa coefficient. Unpaired t-tests, Chi-square tests, and one-way ANOVA were used to determine statistically significant differences between the *TP53* wild type and mutated groups. The diagnostic accuracy of p53 IHC was evaluated by calculating its accuracy, sensitivity, specificity, area under the curve (AUC), positive predictive value (PPV), negative predictive value (NPV), receiver operating characteristic curve (ROC) and area under the curve (AUC) compared to *TP53* tNGS analysis. A two-sided p-value < 0.05 was considered statistically significant.

## Results

### Patient characteristics

The cohort included various MBCL and comprised a (D)LBCL group consisting of Burkitt lymphoma (n = 4), DLBCL, NOS (n = 167), primary cutaneous DLBCL, leg type (PC DLBCL, LT, n = 13), high grade B-cell lymphoma (HGBL, n = 11), primary central nervous system lymphoma (PCNSL, n = 17) and primary mediastinal B-cell lymphoma (PMBL, n = 3). Furthermore, mantle cell lymphoma (MCL, n = 18) and a heterogeneous group of IBCL (n = 76), including NOS (n = 4), follicular lymphoma (FL, n = 52), hairy cell leukemia/lymphoma (HCL, n = 2), lymphoplasmacytic lymphoma (LPL, n = 12), mucosa-associated lymphoid tissue (MALT, n = 8), nodal marginal zone lymphoma (NMZL, n = 17), chronic lymphocytic leukemia (CLL, n = 13) and primary cutaneous follicle center lymphoma (PCFCL, n = 3). All baseline characteristics are depicted in Table 1. The mean age was 63 years (19–95) with a slight male predominance (59%). Analysis was primarily performed on tissue of time at primary diagnosis (n = 252, 71%) and 29% (n = 102) in relapsed or secondary acquired tissue. In the HGBL group, nine patients were classified as double hit genotype (for n = 7 patients translocations MYC + /BCL2 + /BCL6- and 2 cases with translocations MYC + /BCL6 + /BCL2-) and two cases were triple hit genotype.

**Table 1:** Patient and tumor characteristics

Baseline characteristics				
	Total	*TP53* mutated	*TP53* wild type	p-value
Patients (%) Age y, mean (range)	354 63 (19–95)	102 (29) 62 (21–95)	254 (71) 64 (19–89)	0.24
Gender				0.21
Female (%)	145 (41)	36 (25)	109 (75)	
Male (%)	209 (59)	66 (32)	143 (68)	
WHO classification, 2016 (%)				
(D)LBCL (%)	215 (61)	65 (30)	150 (70)	
Burkitt	4 (1)	4 (100)	0	
DLBCL, NOS	167 (47)	52 (31)	115 (69)	
PCDLBCL LT	13 (4)	2 (15)	11 (85)	
HGBL	11 (3)	5 (45)	6 (55)	
PCNSL	17 (5)	2 (12)	15 (88)	
PMBL	3 (1)	0	3 (100)	
MCL	28 (8)	16 (57)	12 (43)	
IBCL (%)	111 (31)	21 (19)	90 (81)	
NOS	4 (1)	1 (5)	3 (95)	
FL	52 (47)	9 (45)	43 (55)	
HCL	2 (2)	1 (50)	1 (50)	
LPL	12 (11)	1 (8)	11 (92)	
MALT	8 (7)	1 (13)	7 (87)	
NMZL	17 (15)	1 (6)	16 (94)	
CLL	13 (12)	6 (46)	7 (54)	
PCFCL	3 (3)	1 (33)	2 (67)	
Tissue				0.11
Primary	252 (71)	66 (26)	186 (84)	
Secondary	102 (29)	36 (35)	66 (65)	

WHO, World Health Organization; (D)LBCL, (diffuse) large B-cell lymphoma; DLBCL, NOS, diffuse large B-cell lymphoma, not otherwise specified; PC DLBCL LT, primary cutaneous diffuse large B-cell lymphoma leg type; HGBL, high grade B-cell lymphoma; PCNSL, primary central nervous system lymphoma; PMBL, primary mediastinal B-cell lymphoma; MCL, mantle cell lymphoma; IBCL, indolent B-cell lymphoma; NOS, not otherwise specified; FL, follicular lymphoma; HCL, hairy cell lymphoma; LPL, lymphoplasmacytic lymphoma; MALT, mucosa-associated lymphoid tissue; NMZL, nodal marginal zone lymphoma; CLL, chronic lymphocytic lymphoma; PCFCL, primary cutaneous follicle centre lymphoma

### P53 IHC

P53 IHC staining quality was sufficient for interpreting the staining patterns in all cases. In the p53 IHC assessment, the two hematopathologists were discordant in sixteen cases, most often between the categories wild type and overexpression (n = 11) vs wild type and complete absence (n = 5). Consensus was achieved through shared evaluation in all cases. The resulting Cohen’s kappa correlation coefficient was 0.88, indicating a strong level of agreement. Wild type p53 expression was observed in 267 cases (75.4%), while an abnormal complete absence pattern was observed in twenty cases (5.7%), and an overexpression pattern was observed in 67 cases (18.9%). All our overexpression cases showed > 50% of tumor nuclei with high-intensity expression of p53 IHC.

### *TP53* assessment by tNGS

**Fig. 2:**
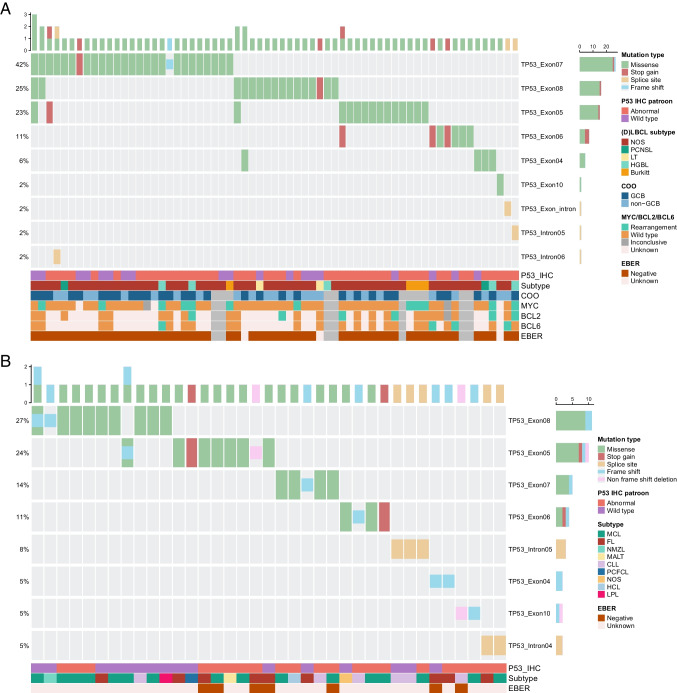
Tumor and molecular characteristics of all *TP53* mutated cases. No pattern with discordant p53 wild type IHC could be identified. **A**: (D)LBCL. **B**: MCL and IBCL. IHC, immunohistochemistry; COO, cell of origin; EBER, Epstein-Barr virus encoded ribonucleic acid; (D)LBCL, diffuse large B-cell lymphoma; NOS, not otherwise specified; PCNSL, primary central nervous system lymphoma; LT, leg type; HGBL, high-grade B-cell lymphoma; GCB, germinal center type; MCL, mantle cell lymphoma; FL, follicular lymphoma; NMZL, nodal marginal zone lymphoma; MALT, mucosa-associated lymphoid tissue; CLL, chronic lymphocytic lymphoma; PCFCL, primary cutaneous follicle centre lymphoma; NOS, not otherwise specified; HCL, hairy cell lymphoma; LPL, lymphoplasmacytic lymphoma

Out of the 354 patients, 102 (29%) had a *TP53* mutation, while 252 (71%) had a wild type status. No significant differences were observed between the mutated and wild type groups in baseline characteristics (Table 1). *TP53* mutations were most commonly observed in (D)LBCL (n = 65), followed by the IBCL group (n = 21), and MCL (n = 16). Overall, most patients had one pathogenic variation in *TP53* (n = 91), nine patients had two different pathogenic mutations and one patient had four different mutations. Using the current tNGS approach, there were no *TP53* gene deletions detected in this cohort. In the *TP53* mutated (D)LBCL group, the most frequent type of mutation observed was a missense mutation (87%), followed by nonsense (8%), splice site (4%) and frame shift mutations (1%). Exon 7 (39%), exon 8 (21%), exon 5 (20%), and exon 6 (9%) were the most common locations of mutation. In MCL (n = 16, 16%), missense mutations (69%) were the most frequent, followed by frameshift (12%), splice site (12%), and nonsense mutations (6%). Exon 8 (44%) and exon 5 (19%) were the most affected locations. The mutation type distribution in IBCL was slightly different, with missense (50%), frameshift (29%), and splice site mutations (13%) being the most frequent types observed. The least frequent types were non-frameshift deletion (8%) and nonsense (4%) mutations. Exon 5 (33%) and exon 8 (17%) were the most affected locations in IBCL. The exon location did not differ significantly between the three groups (p = 0.122). However, the type of mutation was significantly different between LBCL, MCL and IBCL (p = 0.001). Details are presented in a *TP53* mutation oncoplot (Fig. 2).

### Diagnostic performance of p53 IHC

**Fig. 3:**
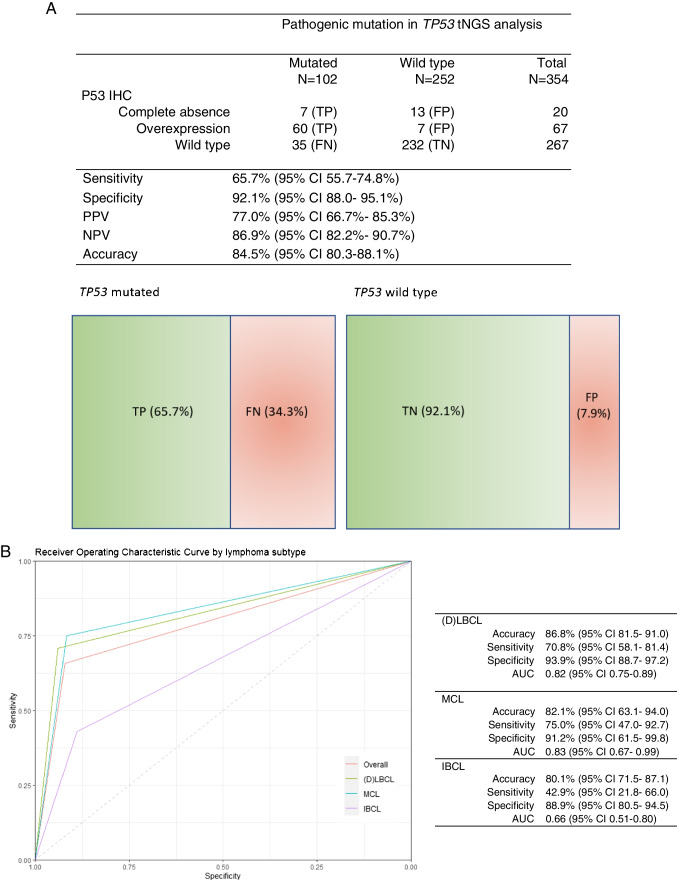
**A** Correlation p53 immunohistochemistry pattern and *TP53* mutational analysis, tNGS, targeted next generation sequencing; IHC, immunohistochemistry; TP, true positive; FP, false positive; FN, false negative; TN, true negative; CI, confidence interval. **B** Diagnostic performance of p53 immunohistochemistry by lymphoma subtype with corresponding ROC curves, IHC, immunohistochemistry; (D)LBCL, diffuse large B-cell lymphoma; MCL, mantle cell lymphoma; IBCL, indolent B-cell lymphoma; AUC, area under the curve

Within the *TP53* mutated group (n = 102, 29%), concordant results with the immunohistochemical assay were found in 67 cases (65.7%). An overexpression pattern was observed in 60 cases (58.9%) of which 54 were missense mutations (90%), one frame shift (2%) and one non frame shift deletion (2%). Two cases had both a missense and nonsense mutation (3%), one case a missense and splice site (2%) and one case a missense and frame shift (2%). A complete absence pattern was seen in seven cases (6.9%), of which six had a truncating mutation. Three cases were splice site mutations (43%), two frame shifts (29%) and one nonsense (14%). One of these cases had a missense mutation (14%). The remaining 35 discordant cases (34.3%) had a false negative p53 wild type staining pattern, despite a confirmed *TP53* mutation. 20 cases in this discordant group had missense mutations (57%), five a nonsense mutation (14%), four a frame shift (11%), four splice sites (11%) and one a frame shift deletion (3%). One case displayed both a missense and frame shift (3%). In the *TP53* wild type group (n = 252, 71%), a concordant wild type p53 IHC pattern was observed in 232 cases (92.1%). In contrast, thirteen cases (5.2%) showed a false positive complete absence staining pattern and seven cases (2.8%) an overexpression pattern, despite the absence of *TP53* mutation. Overall, concordant results between the p53 IHC and tNGS were observed in 299 of 354 cases, resulting in an accuracy of 84.5% (95% CI 80.3–88.1), with a corresponding sensitivity of 65.7% (95% CI 55.7–74.8) and specificity of 92.1% (95% CI 88.0- 95.1). The PPV was 77.0% (95% CI 66.7- 85.3), while the NPV was 86.9% (95% CI 82.2- 90.7), as illustrated in Fig. 3a.

Additional analyses on cases excluding all decalcified samples (n = 313) demonstrated similar overall performance rates. Overall accuracy was 86.0% (95% CI 81.6–74.8) with a corresponding sensitivity of 64.5% (95% CI 53.9–74.2) and specificity of 95.0% (95% CI 91.2–97.5).

### Diagnostic performance per subtype

The diagnostic performance rates per subtype for (D)LBCL, MCL and IBCL were respectively overall accuracy 86.8 (95% CI 81.5- 91.0), 82.1% (95% CI 63.1- 94.0), and 80.1% (95% CI 71.5- 87.1). The sensitivity was 70.8 (95% CI 58.1- 81.4), 75.0% (95% CI 47.0- 92.7), and 42.9% (95% CI 21.8- 66.0). Lastly the specificity was 93.9% (95% CI 88.7- 97.2), 91.2% (95% CI 61.5- 99.8) and 88.9% (95% CI 80.5- 94.5). IBCL demonstrated a similar accuracy and specificity to (D)LBCL and MCL, however the sensitivity of IBCL was lower. In Fig. 3b the ROC curves of LBCL, MCL and IBCL are plotted with the corresponding AUC.

### Discordant cases

**Fig. 4:**
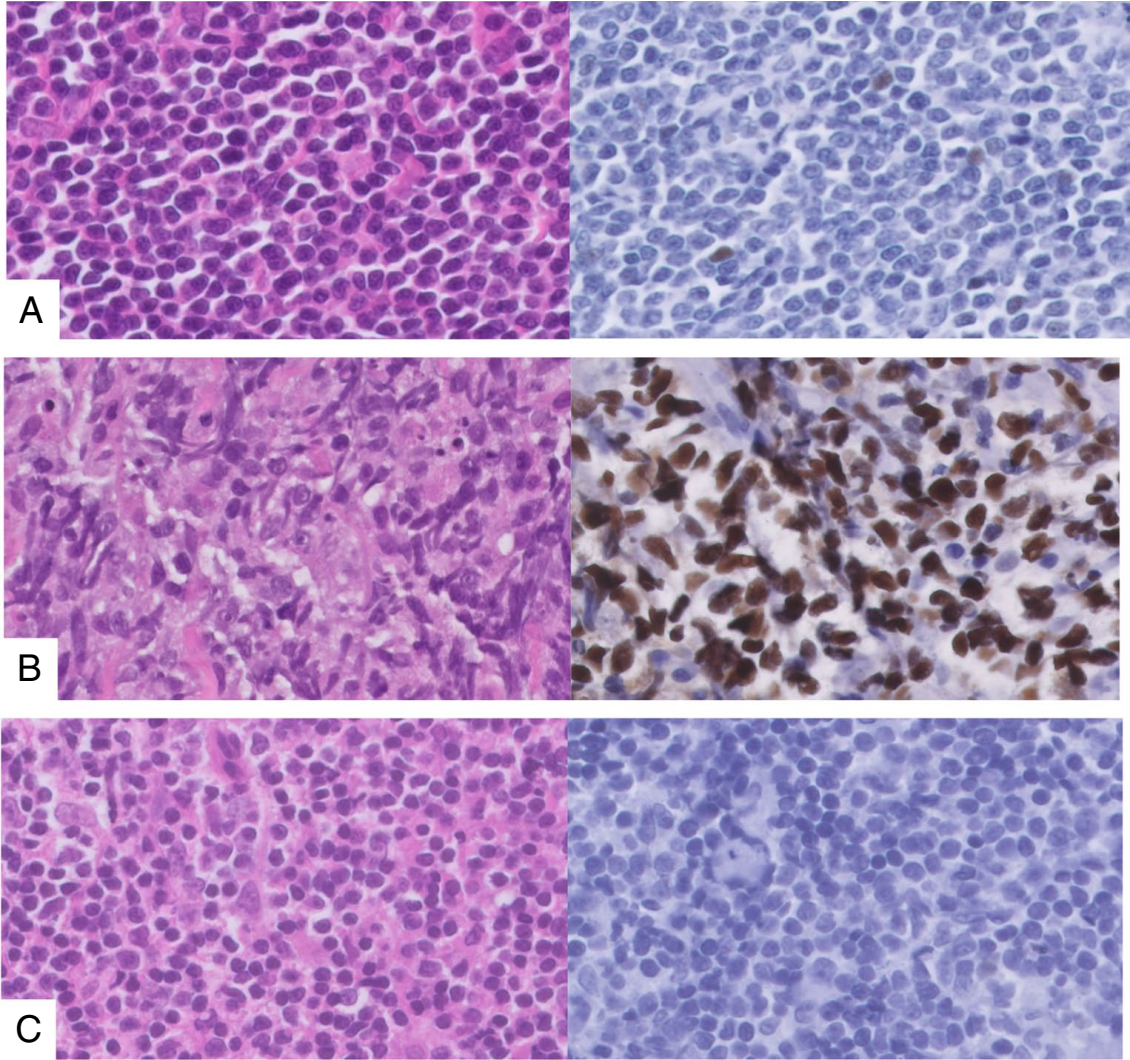
Representative p53 immunohistochemistry of discordant cases, **A** Mantle cell lymphoma (hematoxylin & Eosin × 80) with wild type p53 expression pattern and *TP53* missense mutation, × 80, **B** Diffuse large B-cell lymphoma, not otherwise specified (hematoxylin & Eosin × 80) with abnormal p53 overexpression pattern and no confirmed *TP53* mutation, × 80, **C** Follicular lymphoma (hematoxylin & Eosin × 80) with abnormal p53 complete absence pattern and no confirmed *TP53* mutation, × 80

A total of 55 cases (16%), 20 FP and 35 FN, including (D)LBCL (n = 28, 51%), MCL (n = 5, 9%), and IBCL (n = 22, 40%), demonstrated discordant results between p53 IHC and *TP53* mutational analysis, as indicated in online resource 2. For these cases. needle biopsies (n = 39, 71%) and excisions (n = 16, 29%) were both present, and the tumor percentage was > 50%. Several *TP53* pathogenic variants, sequenced with tNGS panel A or B, were found on various exons, and the variant allele frequency (VAF) was usually > 20%. No significant differences between the false negative and false positive IHC group could be identified in mutation type (p = 0.11), exon location (p = 0.80) or lymphoma subtype (p = 0.10) (Fig. 2 and online resource 3). Upon further analysis of the tNGS data, one LPL case manifested a class IV (likely pathogenic) *TP53* variant, but with an extremely low VAF of 1.6%. Similarly, one other LPL case had a class V (pathogenic) *TP53* variant with a low VAF of 2.4%. For the entire *TP53* wild type group, no gains or losses of *TP53* could be found. Representative examples of discordant cases are shown in Fig. 4.

## Discussion

This study assessed the reliability of p53 IHC as a surrogate marker for *TP53* mutations in a large cohort of MBCL patients. For many years, the implementation of tNGS for routine daily diagnostic procedures for MBCLs placed us in the unique setting to directly compare p53 IHC with *TP53* mutation analyses. Our results showed an overall accuracy of 84.5% for p53 IHC, with a corresponding sensitivity of 65.7% and specificity of 92.1%. Although the specificity was high, the sensitivity was comparatively low, indicating a significant risk of missing *TP53* mutations when using p53 IHC alone, potentially leading to the misevaluation of high-risk patients. Hence, these results indicate that p53 IHC is inadequate as a surrogate marker for *TP53* mutations in the diagnostic workup of MBCL patients. The performance of p53 IHC varied across different subtypes of MBCLs. Specifically, our data suggest that p53 IHC had the least sensitivity for identifying *TP53* mutations in patients with IBCL. This highlights the heterogeneity of MBCLs and the importance of selecting appropriate diagnostic techniques. Therefore, molecular analysis is advised instead of p53 immunohistochemistry in all patients with MBCL, particularly for further advancements of risk-directed therapies based on *TP53* mutation status.

Numerous studies in solid tumors have demonstrated the efficacy of p53 IHC as a surrogate marker for *TP53* mutations, with overall accuracies and sensitivities exceeding 90% [13–15]. Although our high specificity rates are comparable to previous findings in solid tumors, this study shows a lower overall accuracy and sensitivity of p53 IHC in MBCL [13–17]. Studies evaluating the accuracy of p53 IHC in comparison to *TP53* mutation status in lymphomas are scarce and previous studies have primarily centred on (D)LBCL and MCL, rather than the broader MBCL population. While the sensitivity of the IHC was the lowest performance rate in our study, these other studies have found even lower overall sensitivity rates. For instance, in a DLBCL cohort, Xu-Monette et al*.* observed a sensitivity of 48.9% and a specificity of 94.9%, using the same p53 expression cutoff of > 50% (3). Zenz et al*.* reported a sensitivity of 57.1% and specificity of 97.5% in (D)LBCL [26]. In DLBCL, NOS Peroja et al*.* reported a sensitivity of 55.6% and specificity of 90.8% with corresponding positive and negative predictive values of 31.3% and 96.4%, respectively [21]. In MCL, Rodrigues et al*.* reported a sensitivity of 82% and specificity of 100%, which is slightly higher than our findings of 75% and 91%, respectively [27]. In summary, while p53 IHC may serve as an effective surrogate marker for *TP53* mutations in solid tumors, its efficacy in MBCL is less reliable. The reasons for the lower sensitivity of MBCL compared to solid tumors are not yet fully understood. Possible explanations could include technical processing of the tissue or the intrinsic tumor factors. Our study and previous findings highlight the need for alternative or complementary techniques to accurately identify *TP53* mutations and stratify high-risk patients in lymphomas.

In this study, *TP53* mutations were detected in 102 (29%) patients. The prevalence rates of *TP53* mutations in CLL, MCL, and (D)LBCL have been reported to range from approximately 10% to 30%, which aligns with our findings and those of other studies [2, 4, 8]. Similar to our findings, previous studies on (D)LBCL, MCL and IBCL have shown that nonsynonymous or missense mutations in the *TP53* gene are the most frequent, often accounting for over 80% of mutations, followed by nonsense mutations [3, 28–31]. Consistent with other studies, our study found that *TP53* mutations were most often located in exons 5 through 8 in (D)LBCL and MCL. In IBCL, *TP53* mutations are relatively uncommon, ranging from 10 to 20%, and are often associated with poor prognosis and/or transformation [32]. There is limited data on specific exon locations in IBCL, but overall and similar to (D)LBCL, exons 5 through 8 are frequently affected, without a clear subtype-specific pattern [33–35]. Notably, *TP53* mutations in exon 5–8 are found in 94.2% of all tumors, as reported in the IARC database [4].

A major strength of this study is the inclusion of a large patient cohort with a wide variety of MBCL subtypes, including IBCL. This is a retrospective study including 354 patients diagnosed with BCL between 2017–2022. At our center, the implementation of molecular analysis with our in-house developed B-cell tNGS panel in the diagnostic workup of lymphoma patients has expanded greatly over the years. In the beginning (2017–2019), the panel was mainly used for (D)LBCL patients, which explains why some subtypes (e.g., MCL, CLL and FL) are underrepresented in this study. In the subsequent period (2019–2022), tNGS has been applied to all our B-cell lymphoma patients.

Another strength is that both IHC and tNGS were executed and interpreted in a single laboratory, following our standard diagnostic routine, reducing potential technical errors or inter-variabilities between independent hospitals. Incorrect processing, interlaboratory differences, and decalcified tumor tissue can all negatively impact the performance of p53 IHC and result in false negative and false positive staining patterns. Therefore, for interpretation of the IHC it is best if one protocol is used, as performed in our study. A total of 55 cases were discordant between p53 IHC and *TP53* mutational analysis, 20 FP and 35 FN. Of the thirteen cases with complete absence of p53 expression without a mutation, eight were decalcified bone (marrow) biopsies, of which most were LPL (n = 7) and one DLBCL (online resource 2). The suboptimal performance of the IHC in these cases could be attributed to antigen loss during the decalcification process, underscoring a critical limitation of the p53 IHC in routine diagnostic procedures. However, this explanation is not certain, and additional analyses excluding all decalcified samples demonstrated similar performance rates overall and per lymphoma subtype.

There were seven cases with an overexpression pattern, while no *TP53* mutation was detected. No additional mutational analysis was performed to confirm the wild type *TP53* status. DNA and tNGS analysis of all discordant cases were of good quality. Details of all discordant cases are depicted in online resource 3. The tNGS panel used in this study is particularly suitable and widely accepted for the identification of pathogenic mutations, so the likelihood that additional mutational analysis would detect a pathogenic *TP53* mutation is very low. With this NGS panel Copy Number Variant (CNV) analysis was not possible, therefore an undetected *TP53* deletion could be the underlying cause of some of the false positive cases. However, in the case of a *TP53* deletion without an accompanying mutation, the function of the p53 protein is typically preserved with an accompanying wild type IHC expression pattern. In addition, homozygous *TP53* deletions are exceedingly rare.

Our data suggests that wild type cases with strong staining in < 50% of tumor cells cannot be attributed to the relative high number of false negative wild type cases and are also present in the true negative category. Strong staining in tumor cells < 50% would be classified as wild type, however we encountered this expression pattern only once in the false negative category (n = 35), and eight times in the true negative category (n = 232). The other 258 cases had an unambiguous wild type staining pattern with p53 expression in 10–20% of the tumor cells with weak to moderate staining intensity.

Of the seven cases with a complete absence p53 IHC pattern, six had an expected truncating mutation. There was one case with a missense mutation and an unexpected null mutant IHC pattern, without a direct causal explanation.

In recent years, molecular research has gained considerable importance not only in routine diagnostics for lymphoma subtype classification, but also in prognosis and thus patient management. There is a significant research focus on stratifying lymphoma patients who are at high risk of treatment failure with conventional chemotherapy for targeted treatment strategies based on mutational profiles, with *TP53* serving as a critical genetic marker. As mentioned earlier, integrating routine lymphoma diagnostics with prospective clinical data is crucial to comprehensively understand the real-world impact of risk-based profiling and make important advancements in this field [12]. Currently, p53 IHC is a widely available marker that is implemented in most diagnostic pathology departments as part of the routine diagnostic workup. Although p53 IHC is a convenient and well-interpretable marker that is faster and less expensive than tNGS, this study demonstrates that the sensitivity of the IHC alone is insufficient and may result in the misclassification of a high-risk patient. Secondly, despite the finding that a true overexpression pattern of p53 IHC is strongly indicative of a *TP53* mutation (specificity 92%), it is not certain*.* The false-positive rate remains substantial, especially when considering an important treatment decision based on *TP53* mutational status. Therefore, tNGS confirmation over p53 IHC should be strongly considered in MBCL. In cases where additional molecular analysis is not possible or available, p53 IHC alone could be used with caution considering the limitations that were shown here.

In conclusion, this study shows that p53 IHC is an insufficient surrogate marker for *TP53* mutational status in MBCL patients in an unique real-world daily diagnostic setting. By using p53 IHC alone there is a significant risk a *TP53* mutation will be missed, resulting in misclassification of a high-risk patient. Therefore, molecular analysis is recommended over p53 IHC in all patients with MBCL, especially for further development of risk-directed therapies based on *TP53* mutation status.

## Supplementary Information

Below is the link to the electronic supplementary material.

Supplementary file1 (PDF 215 KB)

Supplementary file2 (XLSX 15 KB)

Supplementary file3 (XLSX 31 KB)

## Data Availability

The datasets used and/or analyzed during the current study are available from the corresponding author on reasonable request.
