# Supplementary material for: Real-world routine diagnostic molecular analysis for TP53 mutational status is recommended over p53 immunohistochemistry in B-cell lymphomas
Source: Virchows Arch. 2023 Oct 18;485(4):643–54. doi: 10.1007/s00428-023-03676-6 (PMC11522076; doi:10.1007/s00428-023-03676-6)
Supplement: Supplementary file 1 — Supplementary file1 (PDF 215 KB) [file 428_2023_3676_MOESM1_ESM.pdf]

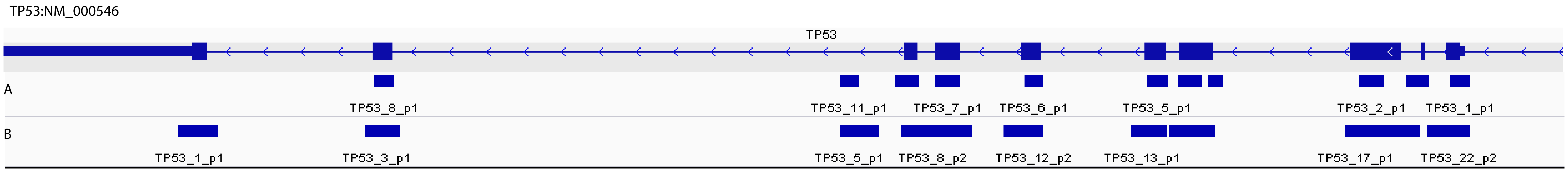

Supplementary figure 1. Graphical presentation of the regions covered by the two tNGS panels.
